# Droplet barcoding for massively parallel single-molecule deep sequencing

**DOI:** 10.1038/ncomms11784

**Published:** 2016-06-29

**Authors:** Freeman Lan, John R. Haliburton, Aaron Yuan, Adam R. Abate

**Affiliations:** 1 Department of Bioengineering and Therapeutic Sciences, California Institute for Quantitative Biosciences (QB3), University of California, San Francisco, California 94158, USA; 2 UC Berkeley - UCSF Bioengineering Graduate program, University of California, San Francisco, California 94158, USA; 3 Integrative Program in Quantitative Biology (iPQB) Biophysics Graduate program, University of California, San Francisco, California 94158, USA; 4 Department of Electrical Engineering and Computer Sciences (EECS), Computer Science Division (CS), University of California, Berkeley, California 94720, USA

## Abstract

The ability to accurately sequence long DNA molecules is important across biology, but existing sequencers are limited in read length and accuracy. Here, we demonstrate a method to leverage short-read sequencing to obtain long and accurate reads. Using droplet microfluidics, we isolate, amplify, fragment and barcode single DNA molecules in aqueous picolitre droplets, allowing the full-length molecules to be sequenced with multi-fold coverage using short-read sequencing. We show that this approach can provide accurate sequences of up to 10 kb, allowing us to identify rare mutations below the detection limit of conventional sequencing and directly link them into haplotypes. This barcoding methodology can be a powerful tool in sequencing heterogeneous populations such as viruses.

## Abstract (web summary)

The ability to accurately sequence long DNA molecules is important across biology. Here, Lan *et al*. report a droplet-based method that barcodes single DNA molecules, allowing the full-length molecules to be sequenced with multi-fold coverage using short-read next-generation sequencing.

## Graphical abstract

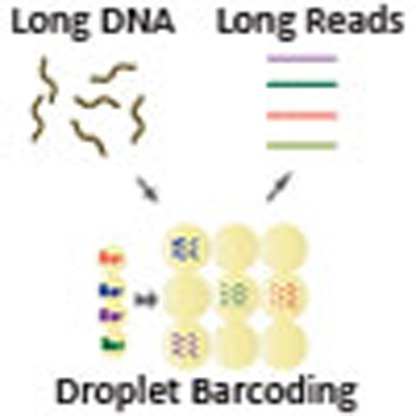

Next-generation sequencing (NGS) has tremendously impacted biomedical research due to its ability to acquire massive amounts of sequence data^1^,^2^. Currently, the most widely adopted sequencing platform produces billions of short (<250 bp) reads at a low cost of ∼$50 per billion bases. However, short NGS reads pose challenges for many applications. For instance, piecing together short reads into long contiguous sequences can be challenging when assembling new genomes, particularly when repetitive sequences are present^3^,^4^. When sequencing metagenomes comprising thousands of species, it is often impossible to assemble the short reads into longer sequences that allow discovery of useful information, such as identification of the species to which a sequence belongs, or detection of gene clusters encoding useful molecules or phenotypes^5^,^6^,^7^. Furthermore, NGS is error-prone, generating an error in every thousand bases; this is often above the rate of biological variation, and consequently, prevents detection of true variants within the cloud of sequencing error^8^,^9^. The ability to obtain massive amounts of long and accurate reads would thus be a major step forward in our ability to characterize genomes accurately, and to study the impact of sequence variation in a variety of systems, such as in rapidly evolving virus populations^10^, rare polymorphisms in human populations^11^, and diverse and uncultivable species in microbial communities^12^.

To obtain longer and more accurate reads, one approach is to directly improve the sequencing instrument^13^,^14^. In addition to providing accurate reads, the instrument must be widely available, easy to use and cost-competitive. Currently, no platform can match short-read NGS in these aspects and as such, short-read sequencers dominate the market. Rather than inventing a new sequencing instrument, an alternative is to synthetically reconstruct long reads from short-read data, leveraging the widespread popularity of short-read NGS. An elegant approach is using unique molecular barcodes, which were first used to detect duplicated NGS reads for error correction, and digital counting of molecules^15^,^16^. To reconstruct long reads using molecular barcodes, long template molecules are broken into short fragments and labelled with ‘barcode' sequences identifying the template from which they originate^17^,^18^,^19^,^20^. All short fragments can then be pooled and sequenced, and fragments of individual templates grouped by barcode. The reads in each group are then used to reconstruct synthetic long reads. Implementations of this approach rely on intramolecular reactions to attach barcodes to the fragments; however, this reaction becomes inefficient for templates above 3 kb. Alternatively, molecules can be physically isolated into wells, followed by fragmentation and barcoding. This approach can theoretically be extended to molecules of any length, but is limited in the number of templates that can be sequenced due to the limitations in throughput of liquid handling in well plates. Throughput can be increased by barcoding multiple templates in each well, but then single-molecule identity is lost^19^,^20^. To enable long and accurate DNA sequencing, an optimal approach would combine physical isolation of molecules with ultrahigh-throughput fluid handling.

In this paper, we describe single-molecule droplet barcoding (SMDB), an ultrahigh-throughput method to barcode long molecules for short-read sequencing. Using droplet microfluidics, we isolate and barcode single molecules in aqueous droplets ∼1 million times smaller than conventional well plates. To validate the method, we sequence a library of known DNA templates of 3–5 kb long and reconstruct long reads fully covering the templates. Furthermore, to demonstrate the ability to sequence large DNA molecules, we apply the method to the *E. coli* genome, obtaining synthetic read-lengths up to 10 kb in length. Finally, to illustrate the power of the method for detecting variants below the detection limit of conventional sequencing, we apply it to a library of β-glucosidase genes mutated by PCR. While SMDB detects 457 SNPs in 81 haplotypes in the library, conventional short-read sequencing detects only one SNP and cannot generate haplotypes. The ability to characterize variants and haplotypes below the inherent detection limit of the sequencer should be powerful for studying systems in which rare variants have an important role, such as in microbial community dynamics and viral quasispecies.

## Results

### Overview of the method

**Figure 1:**
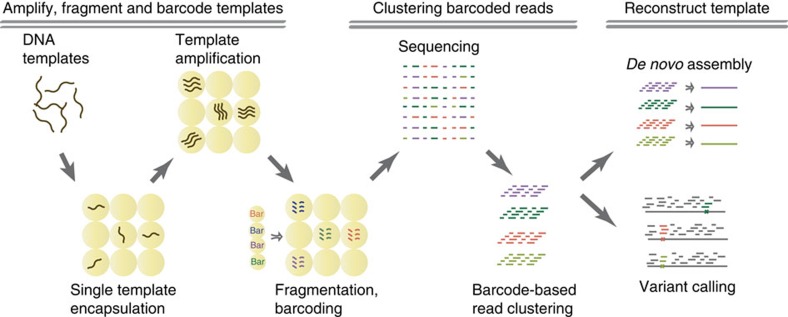
Schematic overview of SMDB. DNA templates are encapsulated into droplets such that most droplets contain zero or one template. Templates are clonally amplified to produce multiple copies in each droplet. Templates are fragmented inside drops, and barcodes are attached to fragments such that each droplet gets a unique barcode sequence. All fragments are sequenced in parallel and resulting reads are clustered based on barcode. Clustered reads are used to reconstruct the sequence or accurately detect SNPs for the template encapsulated in each droplet.

Droplet microfluidics has recently been used to barcode the transcriptomes of single cells^21^,^22^,^23^. In SMDB, we use it to barcode fragments of single DNA molecules, performing all steps of template amplification, fragmentation and barcoding in a microfluidic workflow (Fig. 1). DNA barcodes uniquely tag all reads derived from a template, which allows the reads to be unambiguously clustered to generate a long and accurate consensus sequence for the template.

### Droplet microfluidic workflow for single-molecule barcoding

**Figure 2:**
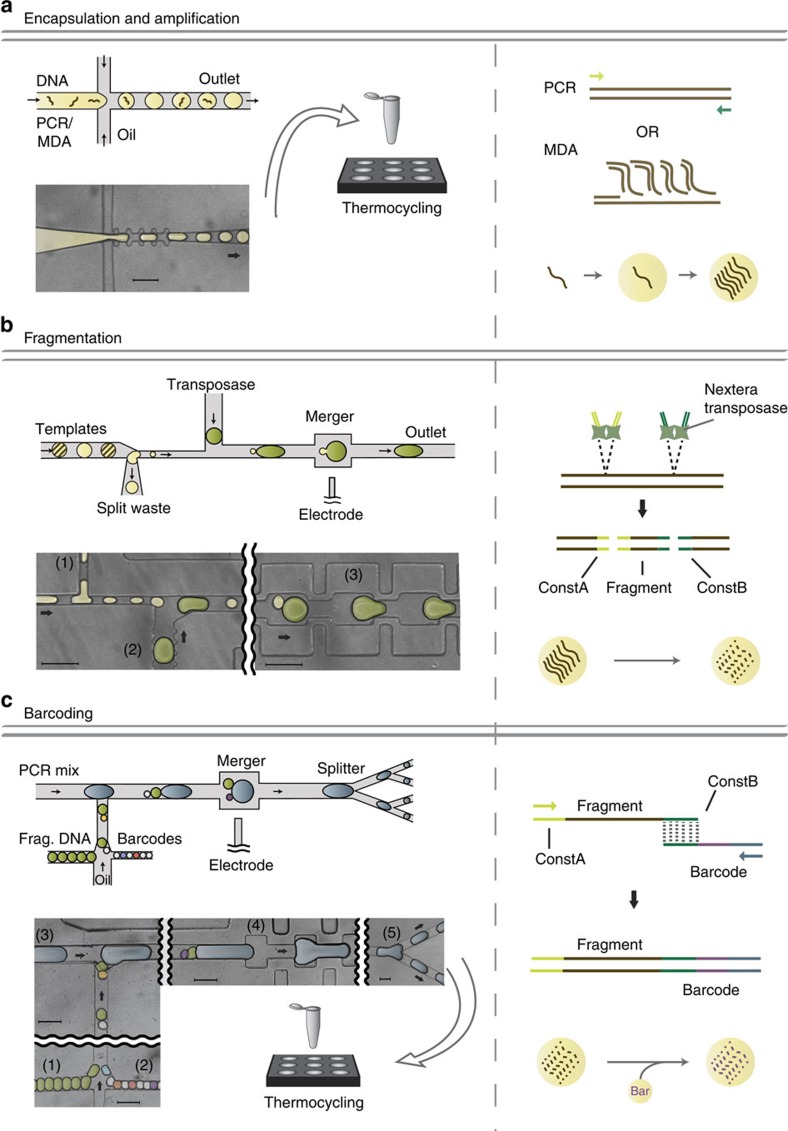
Microfluidic workflow for generating barcoded DNA fragments. Left: schematics and false-colored microscope images of microfluidic devices. Right: schematic of molecular biology reactions occurring inside droplets. (**a**) A flow focus drop maker is used to encapsulate single templates into droplets. Inside droplets, PCR or MDA is used to clonally amplify the single template. (**b**) The splitmerger device is used to add transposases into template drops while achieving a 10 × dilution of the templates. The template droplets are injected on the left side, split at junction (1) so that 1/10th of the droplet continues on to pair with a reagent droplet generated on chip at (2) and the pair merges at the channel widening (3). The transposase reaction inside droplets fragments templates while adding adaptors to each fragment. (**c**) The microfluidic device used for attaching barcodes to DNA fragments. Templates droplets (1) and barcode droplets (2) are injected into the device where they pair with each other and a large PCR reagent droplet generated on chip (3). The three droplets merge at the electrode (4) and are split into smaller droplets for thermal cycling (5). Barcodes are spliced onto fragments by overlap-extension PCR. Scale bars, 100 μm.

We leverage ultrahigh-throughput droplet microfluidics to amplify, fragment and barcode large numbers of individual DNA templates. The first step is to isolate and amplify the template molecules, accomplished by introducing them into a microfluidic flow focus droplet generator that encapsulates them in ∼50 μm diameter droplets of PCR reagent (Fig. 2a). The template concentration is controlled so that ∼1 in 10 droplets contains a single molecule, in accordance with Poisson statistics^24^. The droplets are collected into a PCR tube and thermal cycled for amplification, generating within each droplet a clonal population of the single molecules so that, once fragmented and barcoded, we can obtain multi-fold coverage of each template.

Following amplification, the templates must be fragmented to a length compatible with short-read sequencing. Importantly, fragmentation must be performed while maintaining compartmentalization, to prevent pieces of different templates from mixing before barcodes have been attached. To fragment in the droplets, we use a microfluidic device to add Tn5 transposase into each droplet, which randomly fragments and attaches short sequences to the amplified templates^25^ (Fig. 2b). Because transposases are single-turnover enzymes, an optimal stoichiometric ratio of transposase to templates must be maintained with a 10-fold dilution of the template droplet into the fragmentation droplet. To address this need, we develop a module combining droplet splitting and merging (Fig. 2b and Supplementary Fig. 1). The incoming droplets pass through a junction sampling ∼1/10th of their volume, which is then merged with a new droplet approximately equal to the size of the original droplet. This device accomplishes the necessary tasks of diluting the starting droplet and adding the new reagent, while maintaining the droplet size constant throughout the process. After the transposase is added, the droplets are collected into a syringe and incubated in a water bath at 55 °C for the transposase reaction.

After the templates have been fragmented, the barcodes used to tag fragments belonging to the same template are attached by overlap-extension PCR in the droplets (Fig. 2c). In this reaction, barcode sequences attach to the fragments through regions of sequence homology on the adaptor sequences added by the transposase. This step thus requires merging three droplets: template, barcode and PCR reagent. We design a triple merger device for merging three droplets at once. Improving on the designs of conventional mergers^26^, we concatenate multiple merging junctions, which act independently to achieve robust merging of all three droplets (Fig. 2c and Supplementary Fig. 2). The volumes and reagent concentrations of the droplets are controlled to ensure correct stoichiometry for PCR barcoding. In addition, the channels enable one of each type of droplet to combine in the electro-coalescence junction, shown to the right in Fig. 2c. The resultant droplets are 90 μm spherical diameter and can coalesce during thermal cycling (see Supplementary Note 1 for details on coalescing droplets). To make them more robust, we split the merged droplets into four portions using a splitter^27^. The split droplets are collected into PCR tubes and thermally cycled to attach the barcodes. Even with the small size, ∼10–50% of droplets coalesce (Supplementary Fig. 3a), which is undesirable since it can lead to multiple templates or barcodes in a single droplet, and hence improper barcoding. We therefore remove these droplets using a combination of gravity-induced and pinched-flow fractionation^28^ (Supplementary Fig. 3b and Supplementary Methods). The remaining droplets are chemically ruptured and the DNA contents are purified over a spin column, then size selected to remove free barcodes, resulting in a sequence-ready library.

### Generation of barcode droplets

Uniquely barcoding millions of DNA templates requires tens of millions of ‘barcode droplets', each containing a clonal population of one barcode sequence. To generate these barcode droplets, we individually encapsulate and amplify random barcode molecules using the same technique shown in Fig. 2a (also see Supplementary Fig. 4a). Barcode molecules consisting of random N-mers flanked by constant sequences are chemically synthesized and encapsulated with PCR reagents for amplification. The molecules are loaded at a limiting dilution of ∼1 in 10 droplets. The droplets are thermally cycled, generating within each loaded droplet a clonal population of amplified product; these droplets can then be merged with the template droplets for the barcoding step shown in Fig. 2c. Using this approach, we generate ∼10 million barcode droplets in <1 h for ∼$10 of PCR reagent, which is sufficient to barcode ∼1 million templates in the SMDB workflow.

Because barcode sequences are random, it is possible for two barcodes of the same sequence to label different templates. In *in silico* simulations, we find that the likelihood of this undesirable event is extremely low for barcodes of sufficient length (Supplementary Fig. 4b). During PCR amplification and sequencing of the barcodes, errors and mutations generate a cloud of related sequences around the original barcode sequence. By sequencing our barcode library, we find that the original barcode sequences are on average three Hamming distances from their nearest neighbour, while the sequences within the ‘cloud' of mutated barcodes around each original barcode are, on average, only 1 Hamming distance from their nearest neighbour (Supplementary Fig. 4c). However, the mutated barcodes typically comprise <5% of all reads and do not represent a significant source of inefficiency. To address this issue, we develop an algorithm to cluster mutated barcodes and their parent sequences into a single ‘barcode cluster' (Supplementary Note 2). These barcode clusters represent all fragments that originate from the same template, and thus, are used for template analysis, SNP identification and reassembly.

### Validation of single-molecule barcoding

**Figure 3:**
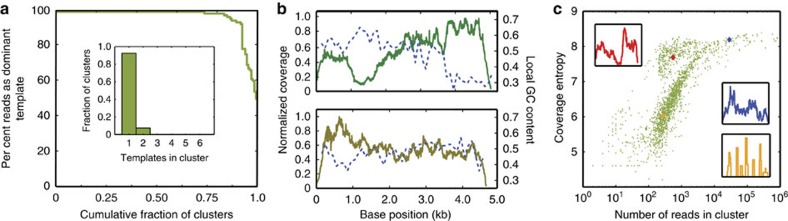
Mapping reads from barcode clusters to the known template references. (**a**) Cumulative distribution of barcode clusters based on per cent of reads that map to the dominant template. The majority of clusters contain reads mostly from a single template. Inset: the number of templates with >10% mapping reads in each barcode cluster is counted and plotted as a histogram. (**b**) Aggregate coverage of two randomly chosen templates for all barcode clusters with corresponding local GC content (dashed line). See Supplementary Fig. 5 for the corresponding plots for all templates. (**c**) All barcode clusters plotted based on coverage entropy and number of reads in each barcode cluster. Each point represents one barcode cluster. Insets: coverage distribution for the individual barcode clusters denoted in corresponding colour on the main plot; *Y* axis: normalized coverage, *X* axis: base position.

A key property of SMDB is its ability to barcode single molecules, which greatly simplifies bioinformatic analysis since all reads in a given cluster are known to originate from only one template. To validate that SMDB indeed barcodes single molecules, we apply it to a library of eight templates from 3 to 5 kb long (for details on known template library, see Supplementary Methods). Because only one-tenth of barcode droplets contain barcodes, we expect only one-tenth of encapsulated templates to be barcoded. Starting with ∼1 M template droplets encapsulated at one in ten droplets containing templates, we expect a theoretical yield of ∼10,000 barcoded templates. Practically, the yield of sequenced templates would be lower due to the sample losses incurred during the start-up of microfluidic devices and during the removal of coalesced droplets. Sequencing the library, we obtain ∼10 million reads using a MiSeq 2 × 250 run, yielding 3,563 clusters, which represents ∼35% of theoretical yield. For perfect barcoding of single molecules, all reads in all clusters should map to only one template. Aligning reads from each cluster to the eight reference sequences, we calculate for each barcode cluster the fraction of reads mapping to the dominant template, defined as the single (out of eight possible) template to which the majority of reads in a cluster map (Fig. 3a). We find that >90% of clusters contain >90% reads mapping to the dominant template. Nevertheless, we observe a low background of <2% of reads mapping to the non-dominant template in less than half of the barcode clusters, which we attribute to mis-tagging, a phenomenon often observed in barcoded sequence libraries prepared in well plates, and thought to originate from chimeric PCR products generated during library amplification and sequencing^29^. Since many barcode clusters contain some degree of non-dominant template reads, we define clusters containing >90% dominant template as single-template clusters. The overwhelming majority (∼90%) of clusters are single-template clusters (Fig. 3a, inset). Instances of multiple templates in the same barcode cluster are infrequent, and consistent with the rate of co-encapsulation expected by Poisson statistics (see Supplementary Note 3 for details). Multiple-encapsulations can be reduced by lowering template concentration, which reduces the instances of multiple templates in the same barcode clusters at the expense of barcoding throughput.

The ideal sequencing data provides full-length, high-accuracy coverage of all templates in the sample. However, bias in sequencing can yield excessive coverage in certain regions and insufficient coverage in others. To investigate whether our approach is susceptible to such bias, we plot the coverage distribution for each template (Fig. 3b and Supplementary Fig. 5). We observe systematic coverage bias for all templates, much of which correlates with local GC content, and hence, is likely the result of the PCR amplification of the libraries for sequencing^30^. We also observe decreased coverage at the ends of templates, a known bias of transposase fragmentation^25^. Thus, the primary forms of bias in our data are the same as those observed in standard NGS, and result from the same sources.

To quantify how bias affects coverage, we define the coverage entropy as the informational entropy of the coverage distribution for each barcode cluster (see Supplementary Note 4 for discussion on coverage entropy). Clusters with high-coverage entropy exhibit flat distributions with uniform coverage, while the clusters with low-coverage entropy exhibit ‘peaky' distributions with non-uniform coverage. Consequently, coverage entropy is a good predictor of whether a cluster contains sufficient information to reassemble a template, and is thus an overall good metric for coverage uniformity (Supplementary Fig. 6a). Plotting the coverage entropy of each barcode cluster against the number of reads contained within it, we observe two populations, one in which entropy saturates rapidly with coverage (upper left) and another in which entropy rises more slowly (Fig. 3c). The clusters where entropy rises slowly with number of reads are more biased, and therefore require more sequencing to obtain the requisite information for assembly. On the basis of our results, an entropy >7 is required for successful assembly (Supplementary Fig. 6a). This corresponds >100 reads in the barcode cluster (Fig. 3c). Therefore, one measure for the efficient utilization of sequencing reads is the number of barcode clusters with >100 reads obtained for a fixed amount of total sequencing reads used (Supplementary Fig. 7). While more sequencing produces more viable barcode clusters, exhaustively sequencing the library results in inefficient utilization of reads.

### SMDB detects rare SNPs and captures haplotypes

An important application of NGS is to detect rare single-nucleotide polymorphisms (SNPs) in heterogeneous populations, such as viruses, cells or human beings^8^,^10^,^31^,^32^. Characterizing that SNPs are physically linked on the same template, called haplotyping, is important for understanding how multiple variants at distant loci can contribute to a given phenotype. However, performing these tasks with conventional NGS is often extremely challenging or impossible due to the inability of the short reads to span multiple SNPs. Moreover, standard NGS is error-prone, generating one error in every ∼1,000 bases; this prevents confident detection of rare variants without accepting a large proportion of false-positives^8^,^9^,^33^. To enhance sensitivity, known patterns of error production can be modelled and used to correct data, providing modest improvements^8^. Molecular techniques can greatly increase sensitivity to detect rare SNPs but reduce read length even further^34^.

**Figure 4:**
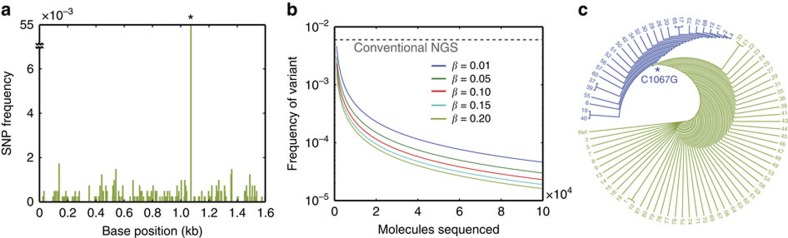
Calling SNPs and haplotypes of single templates from barcode clusters. (**a**) Frequency of SNPs detected at each position in the template. *Indicates the C1067G SNP that can also be detected without barcodes. (**b**) Limit of detection for SMDB for a given number of molecules sequenced. *β* denotes the expected type II error (probability of not detecting the variant). Dashed line represents limit of detection for conventional NGS^29^. (**c**) Phylogenetic tree constructed using consensus sequences generated by the SNP calls. The C1067G mutation and all its derivatives are highlighted in blue. Each node represents a new mutant.

SMDB is able to confidently detect rare SNPs because each molecule is sequenced to great depth, allowing reads to be ‘averaged together' to obtain an accurate consensus for every base. To demonstrate this, we generate a population of DNA templates via 35 cycle PCR of a bacterial plasmid extracted from a culture grown from a single colony. In this population, every sequence shares significant homology, but rare variants exist. Variants like these can have important biological consequences, such as allowing HIV to evolve drug resistance or the development of rare alleles that increase risk for disease in human populations^11^,^33^. We sequence the population using SMDB on a MiSeq 2 × 150 run, obtaining 4.6 million reads in ∼6,000 barcode clusters. Because each barcode cluster represents fragments amplified from a single molecule, we expect a fraction of the fragments—and therefore reads—to contain amplification errors. In the worst case scenario where an error is made in the first round of amplification, we expect ∼50% of the reads to be erroneous for any one position in the sequence. Since these cases are reported as di-allelic SNPs by the SNP-caller, we keep only the mono-allelic SNP calls to ensure the highest accuracy of our mutation calls. We identify 457 high-confidence SNPs in ∼10% of templates, whereas ∼90% of the templates contain no SNPs compared to the reference (Fig. 4a and Supplementary Fig. 8). With the exception of SNP C1067G existing in ∼5.5% of templates, all others are present in <0.1% of the templates, far below the limit of detection for standard NGS. To compare our results to standard SNP calling methods, which do not use barcode information, we call SNPs while disregarding the barcode grouping of reads and detect only the C1067G variant. Hence, SMBD amplifies the sensitivity of sequencing and allows capture of biological information invisible to standard methods. Unlike conventional NGS, the limit of detection of SMDB scales with the number of molecules sequenced and can be easily orders of magnitude more sensitive than conventional NGS (Fig. 4b).

In addition to detecting rare SNPs, SMDB naturally generates haplotypes, which are important for characterizing mutations that have synergistic effects and are broadly relevant from virus evolution to human genetics^35^,^36^. SMDB provides haplotyping information because SNPs that occur on the same template are grouped into the same barcode cluster, allowing haplotypes to be confidently identified for each template. To demonstrate SMDB haplotyping, we plot the haplotypes determined by SMDB in a phylogenetic tree, allowing us to determine the order of mutations that occurred during replication (Fig. 4c). The mutations in the population are generated by replication, and thus, in the absence of selection, ones that occur early in replication exist in a large subset of the progeny. The phylogenetic tree shows that C1067G was the first mutation that arose in the population, consistent with the fact that C1067G mutation is the most abundant SNP.

### SMDB facilitates *de novo* assembly

*De novo* assembly, the process of piecing together short reads into long ‘contigs', is necessary to extract useful information from short reads when a reference sequence is not available, such as when sequencing new genomes or metagenomes^37^,^38^. Despite years of improvement, *de novo* assemblers continue to struggle with datasets comprising multiple sets of highly homologous sequences^18^,^37^,^38^. In some cases, *de novo* assembly is practically impossible because the information needed to uniquely generate a contig spans a length beyond the accessible read length of short-read sequencing. SMDB simplifies *de novo* assembly by ensuring that all reads in a cluster originate from one template, allowing unambiguous assembly of a contig that was previously impossible when all reads from all templates must be considered concurrently.

**Figure 5:**
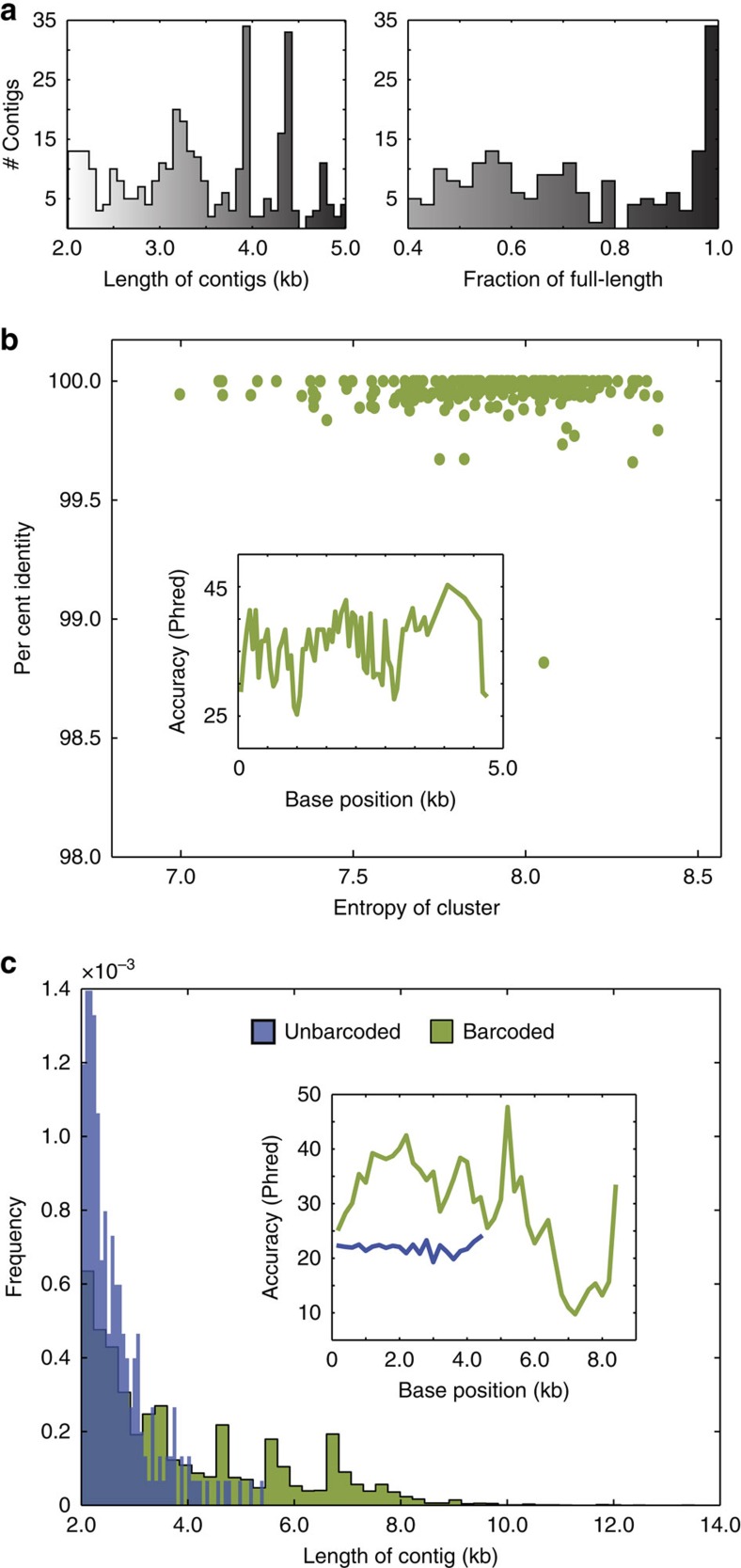
*De novo* assembly of single templates from barcode clusters. (**a**) Distribution of assembled contigs by length (left panel) and fraction of the template covered (right panel). (**b**) Per cent sequence match of each assembled contig to the reference is plotted against the entropy of the barcode cluster that produced the contig. Inset: the accuracy of assemblies for each base position, on a Phred scale, binned by every 50 bp or until the first mismatch to reference if no mismatches are found within 50 bp. (**c**) Distribution of read-lengths of contigs obtained from SMDB of 7–10 kb fragments of the *E. coli* genome. Inset: per-base accuracy of the contigs on a Phred scale.

To demonstrate *de novo* assembly with SMDB, we sequence a test library of known templates 3–5 kb long with a MiSeq 2 × 250, obtaining ∼9 million reads clustering into 2,043 groups. We perform *de novo* assembly on each barcode cluster independently, yielding 245 contigs >2 kb long. The contigs span a range of lengths, and a significant portion of the assembled contigs cover the full length of the templates (Fig. 5a). To account for low-read coverage at the ends of the templates due to biased transposase insertion, we trim the first and last 250 bp of the contigs. The resultant sequences are accurate when compared to the known reference sequences, having an overall error rate of 4.3 × 10^−4^ per base and no detectable structural variations or chimeras. If the errors in the contigs are artifacts of assembly or sequencing, we expect them to be negatively correlated with the coverage entropy of the barcode groups used to assemble them. However, we find contig accuracy is independent of coverage entropy, and rather, depends slightly on position in the contig (Fig. 5b and inset). This is reminiscent of the pattern of SNPs seen in the previous experiment (Fig. 4a), indicating that these are likely rare SNPs rather than errors in the assembled contigs.

Theoretically, any DNA template can be barcoded by SMDB if it can be encapsulated and amplified. However, PCR amplification becomes inefficient for templates longer than 5 kb. To sequence molecules longer than this, we implement multiple displacement amplification (MDA), a non-specific, isothermal method that can amplify whole genomes^39^. We generate fragments of the *E. coli* genome 7–10 kb in length and sequence the resulting library on a MiSeq 2 × 300 run from which we obtain ∼13 million reads clustering into ∼1,000 groups after quality filtering. As expected, *de novo* assembly with barcodes yields significantly longer and more accurate contigs than assembly without barcodes (Fig. 5c and inset). Interestingly, ∼26% of these contigs do not map to the *E. coli* genome, but to other bacterial genomes in the NCBI refseq database, and thus represent contaminating DNA in the library rather than sequencing errors (Supplementary Fig. 6b,c). Thus, SMDB enables sequencing of long templates with arbitrary sequence, but care must be taken to limit contamination.

## Discussion

A challenge when performing molecular biology reactions in droplets is that, often, multiple reagents must be added to the droplets at different times. Since reagent addition always increases the size of the droplets, adding multiple reagents can produce final droplets that are too large to be robustly handled. To perform reagent addition while maintaining droplets at a reasonable size, we have developed a split–merge device that combines droplet splitting with droplet merger^26^,^40^. This device has the unique and valuable property of producing final droplets that are equal in size to the initial droplets; hence, this same device can be used to perform multiple additions on an emulsion while maintaining constant droplet size. The degree of dilution can be adjusted by varying the amount sampled from the split droplet, which is adjusted by controlling the flow rate of the splitting outlet. This obviates the need to construct a unique device with increasing dimensions for each round of reagent addition, and maintains the droplets in the size range that is optimized for handling and incubation. The split–merge device should be valuable when multiple reagent additions must be performed on an emulsion—a task that has thus far been a significant challenge for droplet microfluidic workflows.

The random Poisson encapsulation of templates and barcodes is a source of inefficiency in SMDB, but one that is overcome by leveraging the ultrahigh-throughput nature of droplet microfluidics. To ensure that most templates are paired with a single barcode, barcodes and templates are loaded at ∼1 in 10 per droplet, yielding a single pairing event for ∼1 in 100 droplets. Even with this inefficiency, the throughput of our device enables barcoding of ∼3,500 molecules in ∼15 min. Assuming a modest template length of 5 kb, this is sufficient to cover an *E. coli* genome at ∼5 × coverage. With higher-throughput droplet generation and manipulation, such as emulsification under jetting conditions^41^ and parallelization of channel networks^42^,^43^, it should be possible to increase throughput by an order of magnitude. In addition, the template and barcode emulsions can be sorted to discard empty droplets, which should increase efficiency ∼10-fold by ensuring that every pairing event comprises one of each component with no wasted droplets.

Encapsulation of templates into small volumes reduces amplification bias during PCR but also limits the amount of DNA generated for each barcoded template. Therefore, the number of starting templates is directly correlated with the amount of DNA obtained at the end of the workflow. We have empirically determined that >10,000 productive droplets are required to provide the minimum ∼20 nanomoles for sequencing after accounting for sample loss through the workflow. Although it is possible to additionally PCR amplify lower yield libraries, this results in more bias, yielding uneven coverage of templates, and uneven distribution of reads into barcode groups.

Droplet microfluidic workflows have been successfully adapted into non-microfluidic labs through collaboration with labs with microfluidic expertise^21^,^22^. For labs interested in adopting SMDB, we suggest collaborating with a droplet microfluidics lab, because although the fabrication and operation of the microfluidic devices is straight forward, the handling of droplets outside of devices is quite nuanced. Dolomite, a company dedicated to providing off-the-shelf and custom designed droplet microfluidic devices for research, is also an excellent resource for implementing droplet microfluidics workflows into the lab.

New technologies for sequencing DNA while retaining long-range information are becoming available^20^,^44^. While these technologies share some similarity to ours, there are critical differences that make each approach better or worse for different applications. For example, recent methods that encapsulate many template molecules in each droplet provide very high throughput and are an inexpensive solution for barcoding large amounts of DNA, but the resulting sequence data cannot be deconvoluted back to single molecules since within each barcode cluster (droplet) many templates of different sequences exist. This may be acceptable for applications in which the templates are highly dissimilar or in which single-molecule resolution is not required, but in others it may prove problematic. In particular, for samples in which the molecules share significant homology but small sequence differences are biologically relevant, such as when studying viral diversity and evolution, these technologies are ineffective and the SMDB approach is better suited. A similar technology specifically targeted to sequence human genomes is available and therefore applications of SMDB to human genome sequencing are not investigated^45^.

We have applied SMDB to the barcoding of single DNA molecules from virus and microbial genomes, but the principle of encapsulating and barcoding nucleic acids in microfluidic droplets is broadly applicable. For example, droplet microfluidics has been used to encapsulate, lyse, and amplify single viruses and cells^46^,^47^. The SMDB workflow we describe here could be combined with these methods to barcode the genomes of these organisms, to perform whole-genome single virus or cell sequencing. This could make the barcoding workflow valuable for characterizing genetic reassortment in seasonal influenza. Indeed, while barcoding up to ∼10,000 single entities is immediately practical with the methods we describe, if single cells rather than long templates were to be barcoded, the number of individual genomes that can be sequenced is limited by the sequencing throughput of NGS. Even with the massive capacity available with present-day instruments, it is not enough to fully leverage the throughput of our droplet method. However, as sequencing instruments continue to decrease in cost and increase in throughput, sequencing large barcoded populations of cells and viruses should become practical, impacting applications in which genetic diversity is important, such as in microbial communities.

## Methods

### Microfluidic devices

Photoresist masters are created by spinning on a layer of photoresist SU-8 3025 (Microchem) onto a 3 inch silicon wafer (University Wafer) at 3,000 rpm, then baking at 95 °C for 5 min. Then, the photoresist is subjected to 3 min ultravoilet exposure over photolithography masks (CAD/Art Services) printed at 12,000 DPI. After ultravoilet exposure, the wafers are baked at 95 °C for 10 min then developed for 10 min in fresh propylene glycol monomethyl ether acetate (Sigma Aldrich) then rinsed with fresh propylene glycol monomethyl ether acetate and baked at 95 °C for 5 min to remove solvent. To fabricate the triple merger device, a second layer of photoresist was patterned on top of the first layer after the first ultravoilet exposure to generate a two-layered master. The microfluidic devices are fabricated by curing poly(dimethylsiloxane) (10.5:1 polymer-to-crosslinker ratio) over the photoresist master^48^. The devices are cured in an 80 °C oven for 1 h, extracted with a scalpel, and inlet ports added using a 0.75 mm biopsy core (World Precision Instruments, catalogue no. 504529). The device is bonded to a glass slide using O_2_ plasma treatment and channels are treated with Aquapel (PPG Industries) to render them hydrophobic. Finally, the devices are baked at 80 °C for 10 min to dry the Aquapel before they are ready for use.

### Barcode emulsion

Chemically synthesized barcode oligonucleotides (GCAGCTGGCGTAATAGCGAGTACAATCTGCTCTGATGCCGCATAGNNNNNNNNNNNNNNNTAAGCCAGCCCCGACACT) (IDT) are added at 0.01 pM concentration into a PCR reaction mix containing 1 × NEB Hotstart Phusion polymerase (NEB, catalogue no. M0536L), 2% w/v Tween 20, 2% w/v PEG 6000, 400 nM forward and reverse primers (FL128 CTGTCTCTTATACACATCTCCGAGCCCACGAGACGTGTCGGGGCTGGCTTA) (FL129 CAAGCAGAAGACGGCATACGAGATCAGCTGGCGTAATAGCG). The reaction mixture and HFE 7500 fluorinated oil (3 M) with 2% (w/w) PEG-PFPE amphiphilic block copolymer surfactant (Ran Biotechnologies) are loaded into separate 1 ml syringes and injected at 300 and 500 μl h^−1^, respectively, into a flow-focusing droplet maker using syringe pumps (New Era, catalogue no. NE-501) controlled with a custom Python script (https://github.com/AbateLab/Pump-Control-Program). After collecting the emulsion in PCR tubes, the oil underneath the emulsion is removed using a pipette and replaced with FC-40 fluorinated oil (Sigma Aldrich, catalogue no. 51142-49-5) with 5% (w/w) PEG-PFPE amphiphilic block copolymer surfactant for improved thermal stability (see Supplementary Note 1 for details on thermostability). The emulsion is transferred to a T100 thermocycler (BioRad) and thermally cycled with the following program: 98 °C for 3 min, followed by 40 cycles with 2 °C per second ramp rates of 98 °C for 10 s, 62 °C for 20 s and 72 °C for 20 s, followed by a final hold at 12 °C. SYBR staining using 10 × SYBR GREEN I in HFE 7500 oil is used to quantify encapsulation rate under a fluorescent microscope.

### Generating template droplets

For SMDB using PCR, DNA template molecules are encapsulated and amplified in the same manner as described above, except the primers used are FL178 (CCACTACGCCTCCGCTTTC) and FL179 (CCATCTCATCCCTGCGTGT), and input DNA is a library of long molecules with universal adaptors on either side. Input DNA concentration is adjusted until one in ten droplets are fluorescent under SYBR staining. To construct the library of seven known templates, eight DNA templates are amplified from 5 ng of phage lambda genomic DNA (NEB: N3013S) using 500 nM of primer sets (see oligonucleotides listed in Supplementary Table 1) using 1 × NEB phusion hotstart flex mix (NEB: M0536S) with the following cycling conditions: 98 °C 3 min, 35 cycles of: 98 °C 15 s, 62 °C 30 s, 72 °C 3 min, followed by 72 °C 5 min and optional holding at 12 °C overnight. The PCR products are gel-extracted using 1% agarose gel and Zymo gel extraction kit. To attach constant sequence adaptors to all the fragments, 100 ng of gel-extracted amplicons are added to an adaptor ligation mix of: 1 μM adaptors, 0.20 mM dNTPs, 0.5 μl (60 units) of Bst 2.0 polymerase warmstart (NEB: M0538M), 2.5 μl T4 DNA ligase from the quick ligation kit (NEB: M2200S), 1 × ligase buffer from the quick ligase kit. The reaction is incubated at 25 °C for 15 min then 65 °C for 10 min for heat inactivation, then DNA is purified using the Zymo DNA concentrator kit. The concentration of resulting DNA is quantified using the bioanalyzer high sensitivity kit and pooled together at equal molar concentration to generate the eight templates library.

For SMDB using MDA, reactions are performed using REPLI-g single cell kit (Qiagen, catalogue no. 150343). *E. coli* genomic fragments are from *E*. *coli* K12(DH10B) cells purchased from New England BioLabs (catalogue no. C3019H), lysed and purified using PureLink Genomic DNA Mini Kit (Life Technologies, catalogue no. K1820-00). Ten kilobase fragments are gel-extracted following a 10-min digestion with NEBNext dsDNA Fragmentase (NEB, catalogue no. M0348S) of 800 ng DNA and quantified using a NanoDrop (Thermo Scientific). The fragmented input DNA is incubated with 3 μl Buffer D2 and 3 μl H_2_O for 10 min at 65 °C. After stopping by adding 3 μl stop solution, a master mix comprising nuclease-free H_2_O, REPLI-g reaction buffer, and REPLI-g DNA polymerase is added. The MDA reactions are then emulsified in the manner described above and incubated at 30 °C for 3 h then 70 °C for 20 min for heat inactivation.

### Fragmentation of templates in droplets

Droplets containing amplified templates, a Nextera Transposase reaction mixture composed of 1 × TD buffer, 2% w/v Tween 20, 2% w/v PEG 6000, and 1/10 volume of TDE from Nextera Kit (Illumina, catalogue no. FC-121-1031, or purified in lab as described^49^), deionized water, HFE 7500 with 2% w/v EA surfactant and 2 M NaCl are loaded into 1 ml syringes (BD scientific) and connected to the split-merge microfluidic device (Supplementary Fig. 1). The electrode is connected by clipping the output of a cold cathode fluorescent inverter connected to a DC power supply (Mastech) to the needle of the electrode syringe using an alligator clip. Setting a voltage of 2.0 V at the power supply results in a ∼2 kV AC at the electrode, which causes droplets close to the electrode to merge. The resulting emulsion is collected in a 1 ml syringe and incubated at 55 °C for 10 min and then 70 °C for 20 min in large water baths.

### Barcoding of fragmented templates

Fragmented template droplets, barcode droplets and a PCR mixture composed of 1 × Invitrogen Platinum Multiplex mix (ThermoFisher, catalogue no. 4464268), 400 nM Primers FL127 (AATGATACGGCGACCACCGAGATCTACACTCGTCGGCAGCGTC) and FL129 (CAAGCAGAAGACGGCATACGAGATCAGCTGGCGTAATAGCG), 1 in 50 dilution of the NT buffer from the Nextera XT Kit (0.2% SDS) (Illumina, catalogue no. FC-131-1024), 1% Tween 20 w/v, 1% PEG 6000, w/v, 2.5 U μl^−1^ Bst Polymerase 2.0 Warmstart (NEB catalogue no. M0538S) are loaded into a syringe and injected into the double merger device as shown in Supplementary Fig. 2. The emulsion is collected in a 0.5 ml thin-walled PCR tube, and the oil is replaced with FC-40 with 5% w/v EA surfactant before thermal cycling at: 65 °C for 5 mins, 95 °C for 2 mins, then 25 cycles at 2 °C/s ramp rates of 95 °C for 15 s, 60 °C for 1 min, 72 °C for 1 min, and then 72 °C for 5 min followed by optional 12 °C hold overnight. After thermal cycling, the oil is replaced with HFE 7500 with 2% w/v EA surfactant, then loaded into a syringe injected into a pinched-flow fractionation device to remove large droplets as shown in Supplementary Fig. 3b. After removal of large droplets, the emulsion is broken by adding 20 μl of 1H,1H,2H,2H-Perfluoro-1-octanol (Sigma Aldrich, catalogue no. 370533) and brief centrifugation in a micro-centrifuge. The aqueous top phase is collected and DNA is purified using a Zymo DNA concentrator kit.

### Final library amplification

Overall, 2 ng of the barcoded library is added to a PCR mixture containing 1 × Phusion master mix and 400 nM of primer FL127/129 and thermal cycled as follows: 98 °C 3 min, and 10 cycles of 98 °C 10 s, 62 °C 20 s, 72 °C 1 min, 72 °C 5 min. The resulting DNA is loaded into a Blue Pippin (Sage Biosciences) 100–600 bp cassette to extract DNA from 300–700 bp range to remove free untagged barcodes. The resultant DNA is concentrated using a Zymo DNA concentrator kit, quantified using the Bioanalyzer high sensitivity DNA chip (Agilent) and sequenced on the MiSeq using a custom index primer (FL166).

### Bioinformatics analysis

Barcodes are clustered using a python program dfs clustering (Supplementary Note 2), which uses raw Miseq fastq files and outputs barcode clusters and the IDs of their associated reads along with quality control metrics. Barcode clusters containing <500 reads are removed because they contain too few reads for analysis. For SNP calling, reads from each barcode cluster are mapped onto the template sequence using Bowtie 2 (ref. 50)—very-sensitive-local settings and outputted as a SAM file then converted into a BAM file using Samtools. To call SNPs, Samtools v1.2 mpileup is used with options-d 9999-u-V-I and Bcftools call is used with options -c -v to filter only positions that contain SNPs. The SNPs are then filtered for homozygous calls as there should be only one template per barcode cluster. The phylogenetic tree was constructed using consensus sequences generated by the replacing each position of the reference with the SNP called for each barcode cluster. Duplicate sequences are removed, and then the list of non-redundant consensus sequences are used to generate a phylogenetic tree using the maximum likelihood method in Phylip v3.696. For *de novo* assembly, reads from each barcode cluster are written into one file in fasta format with bases lower than Q20 replaced with N. Each fasta file is fed into the IDBA-UD assembler v1.1.1 (ref. 51) with parameters—mink 20—maxk 120—step 20—min_contig 2000—min_count 1—max_mismatch 3.

### Data availability

Sequencing data generated from SMDB of the seven templates control and *E. coli* genomic fragments libraries are available at the Sequence Read Archive (SRA) under accession code SRP072529.

## Additional information

**Accession codes**: Sequencing data generated from SMDB of the 7 templates control and *E. coli* genomic fragments libraries are available at the Sequence Read Archive (SRA) under accession code SRP072529.

**How to cite this article:** Lan, F. *et al*. Droplet barcoding for massively parallel single-molecule deep sequencing. *Nat. Commun.* 7:11784 doi: 10.1038/ncomms11784 (2016).

## Supplementary Material

Supplementary Information Supplementary Figures 1-8, Supplementary Table 1, Supplementary Notes 1-4 and Supplementary Methods.
