# Supplementary material for: Droplet barcoding for massively parallel single-molecule deep sequencing
Source: Nat Commun. 2016 Jun 29;7:11784. doi: 10.1038/ncomms11784 (PMC4931254; doi:10.1038/ncomms11784)
Supplement: Supplementary Information — Supplementary Figures 1-8, Supplementary Table 1, Supplementary Notes 1-4 and Supplementary Methods. [file ncomms11784-s1.pdf]

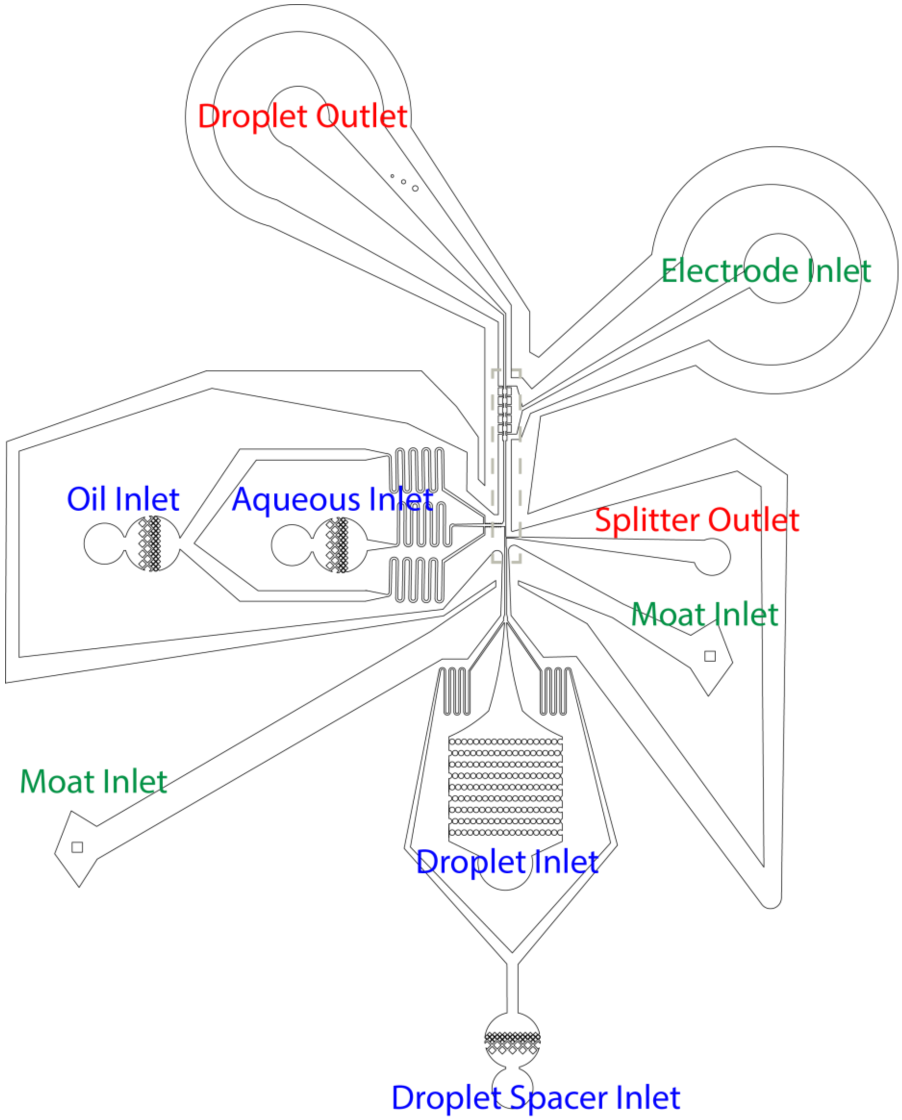

Supplementary Figure 1. Schematic of split-merger microfluidic device used to add transposase to template drops for fragmentation. Inlets are labelled in blue, outlets are labelled in red, and static channels labelled in green are filled with 2M NaCl. This device consists of four modules: the droplet inlet and droplet spacer used to reinject drops; the splitter channel used to controllably split a fraction of every drop; the oil inlet and aqueous inlet combining into a droplet maker, and the electrode and moat channels forming a merger channel. The dashed box denotes the area of the device shown in Fig. 2 of the main text. Pump flowrates: oil 700 µL per hr, aqueous 250 µL per hr, droplet 150 µL per hr, droplet spacer 200 µL per hr, splitter 170 µL per hr.

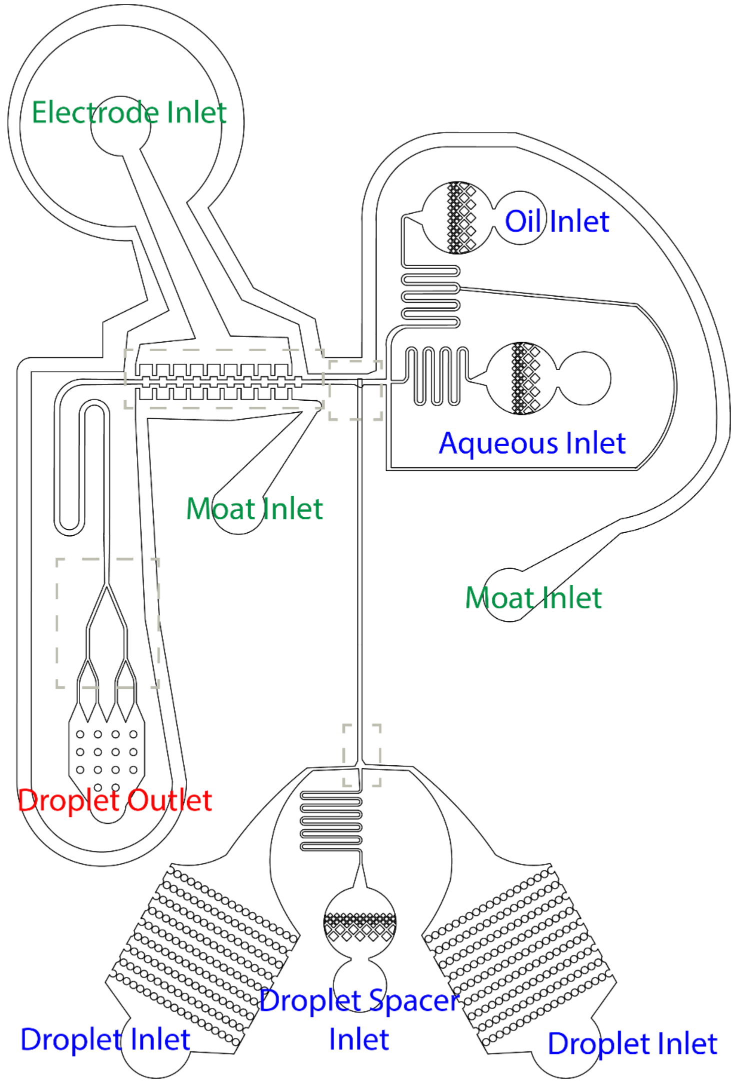

Supplementary Figure 2. Schematic of the barcode merging device which merges two small drops with one large drop then splits the large drop into 4 smaller drops. Inlets are labelled in blue, outlets are labelled in red, and static channels labelled in green are filled with 2M NaCl. This device consists of four modules. The droplet inlets and spacer used to reinject and intercalate two sets of drops. The aqueous and oil inlets to generate large droplets on the device. The moat and electrode channels to merge the droplets. The bifurcations at the outlet to split large droplets into small droplets. The dashed boxes denotes the area of the device shown in Fig. 2 of the main text. Pump flowrates: oil 10000 µL per hr, droplet inlets 50 µL per hr and 70 µL per hr, aqueous 800 µL per hr, spacer 200 µL per hr.

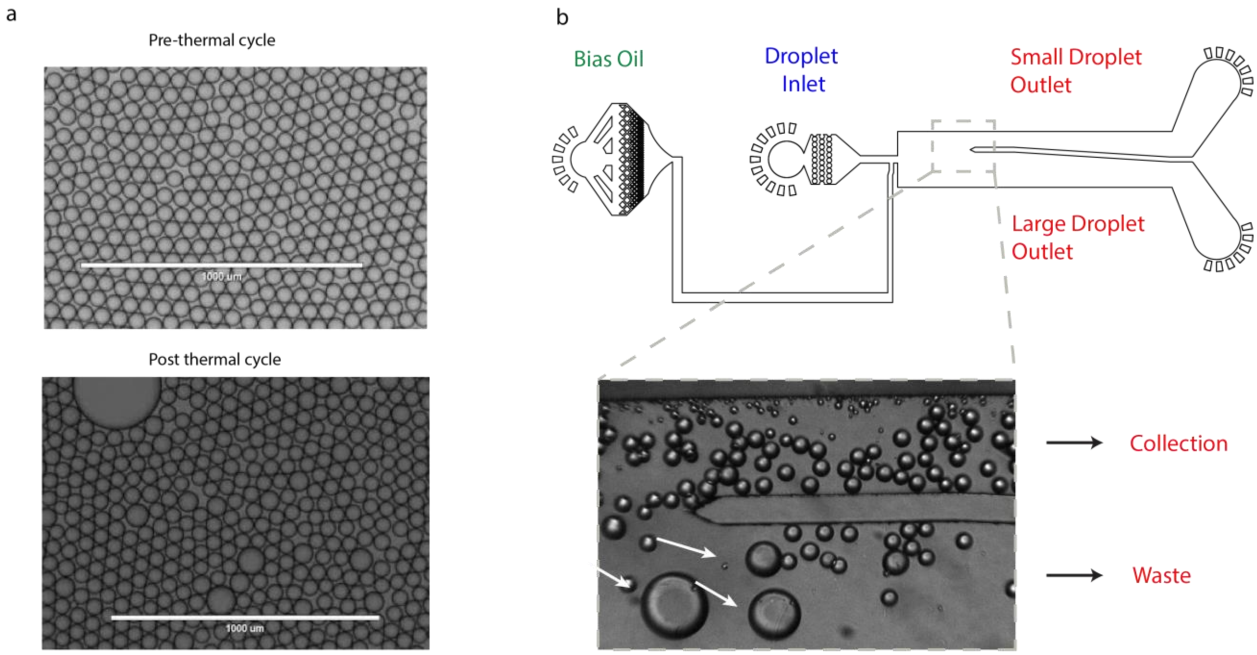

Supplementary Figure 3. The pinched flow fractionation device used to remove large coalesced droplets from an emulsion**.** a) Emulsion collected at the end of the triple merger device before and after thermal cycling, showing coalescence droplets which will be sorted out in the next step. b) Schematic and bright field picture of the pinched flow fractionation device used to remove large droplets. Droplets are injected at 400 µL per hr. HFE7500 oil is injected at 4000 µL per hr. The smaller droplets are collected into tube, while the outlet for large droplets is attached to a syringe filled with water, pulling at 3000 µL per hr. Microscope image of pinched flow fractionation device separating large and small droplets. Arrows indicate large droplets that flow to the lower outlet, separating them from the smaller droplets.

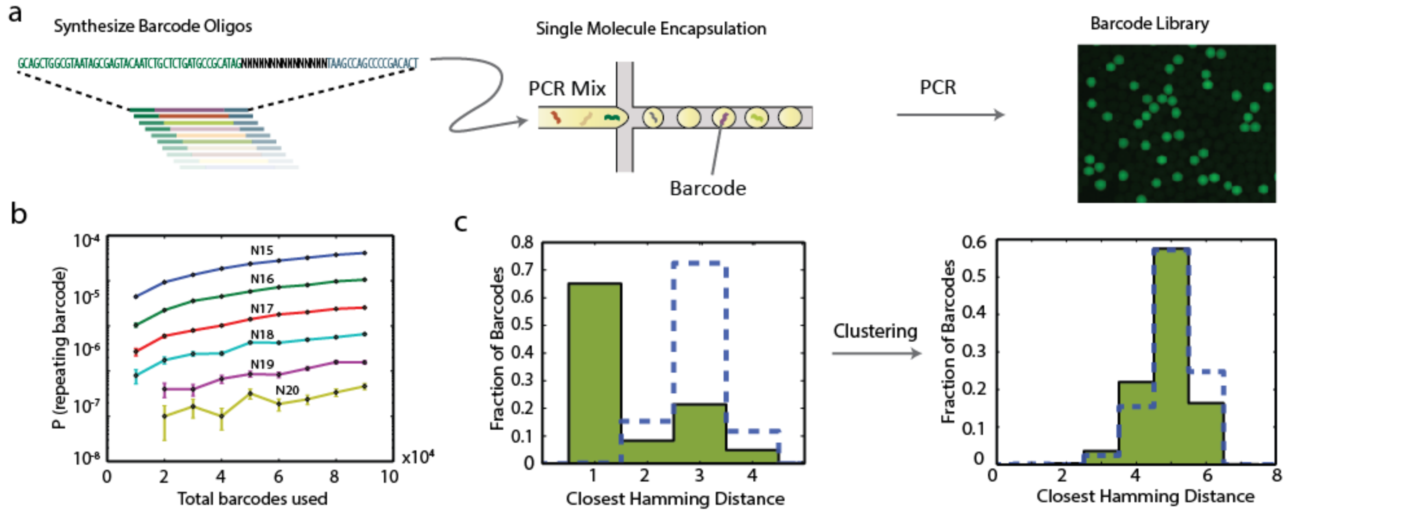

Supplementary Figure 4. Schematic and validation of droplet barcode library. a) Chemically synthesized random *N*mer barcodes are encapsulated into droplets so that most droplets contain zero or one barcode. Inside droplets, each barcode is clonally amplified by PCR, generating droplets that contain zero or many copies of a unique barcode. SYBR staining inside droplets is used to identify droplets that contain barcodes. b) Plot showing the probability of reusing the same barcode for an experiment using a total number of barcodes, for barcodes of 15-20 bps long. Error bars represent the standard error of the mean from 10,000 simulation runs. See supplemental methods for simulation details. c) Empirical data from a SMDB sequencing library. Distribution of Hamming distance of each barcode to its closest neighbor before and after clustering. Error barcodes are 1 Hamming distance away from their closest neighbor while original barcodes are on average three Hamming distances away. Dashed blue line shows the theoretical distribution of Hamming distances given an equal number of randomly chosen barcodes.

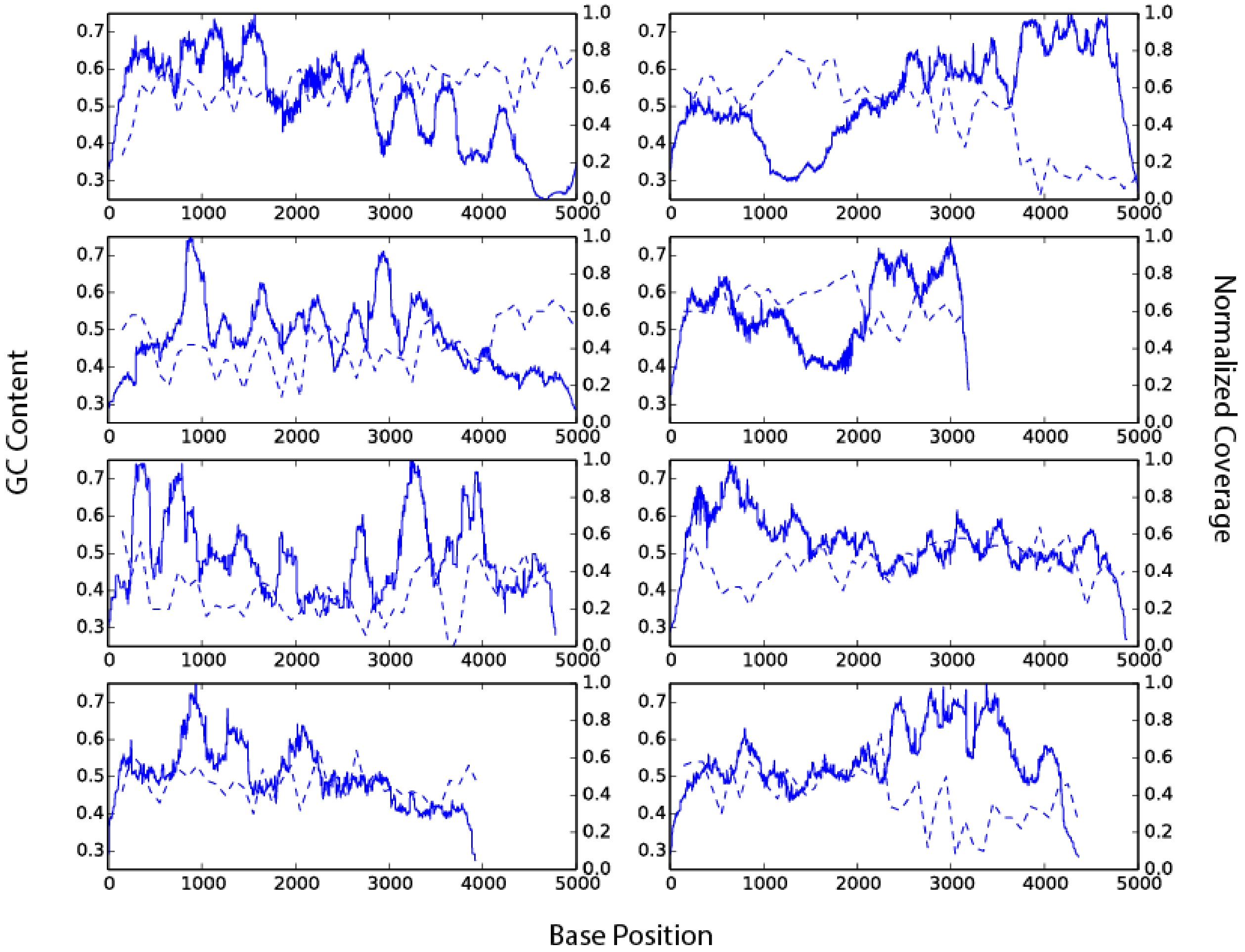

Supplementary Figure 5. Local GC content and aggregate coverage for all 8 templates. Dashed line shows local GC content of the templates plotted on the left axis. Solid line shows the aggregate normalized coverage for each template plotted on the right axis.

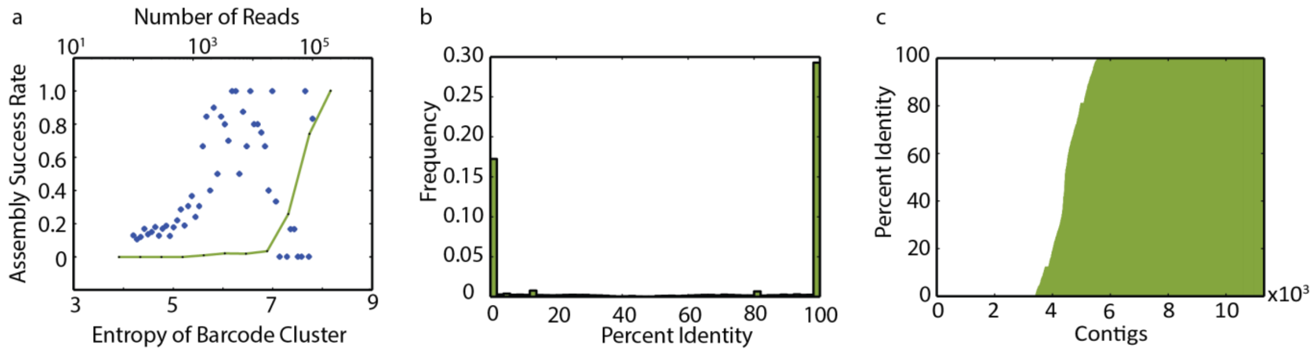

Supplementary Figure 6. Additional metrics on success of *de novo* assembly of barcode clusters a) Correlation between *de novo* assembly success rate and coverage entropy (green line) for the known templates dataset. For comparison, assembly success is also plotted against the number of reads in the barcode cluster (blue dots) showing a weaker relationship. b) Percent identities of contigs assembled from the *E. coli* genomic DNA library compared to the reference *E. coli* genome. c) The same data with each contig represented on the x-axis.

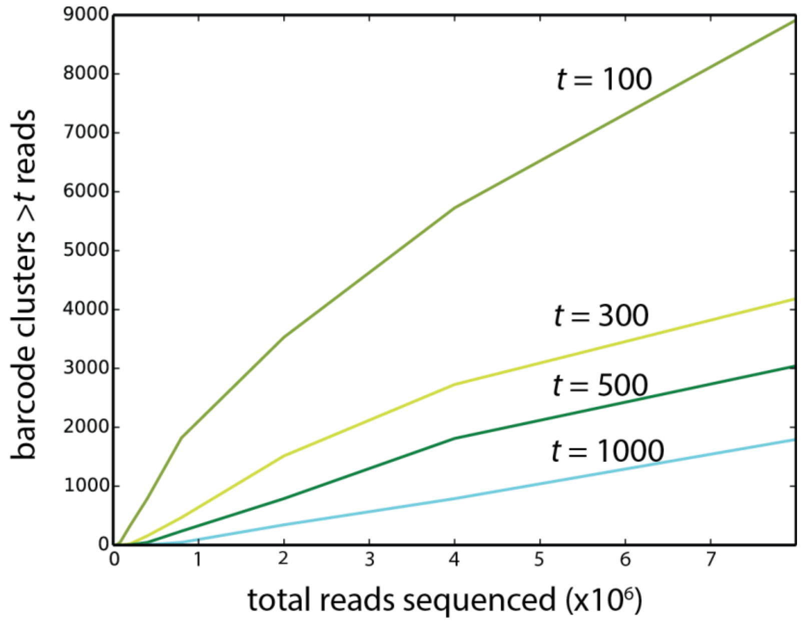

Supplementary Figure 7. Number of barcode clusters containing a minimum number of reads for a given sequencing effort. More sequencing effort results in more barcode clusters containing the minimum number of reads, but the rate of new barcode cluster discovery decreases as more reads are sequenced.

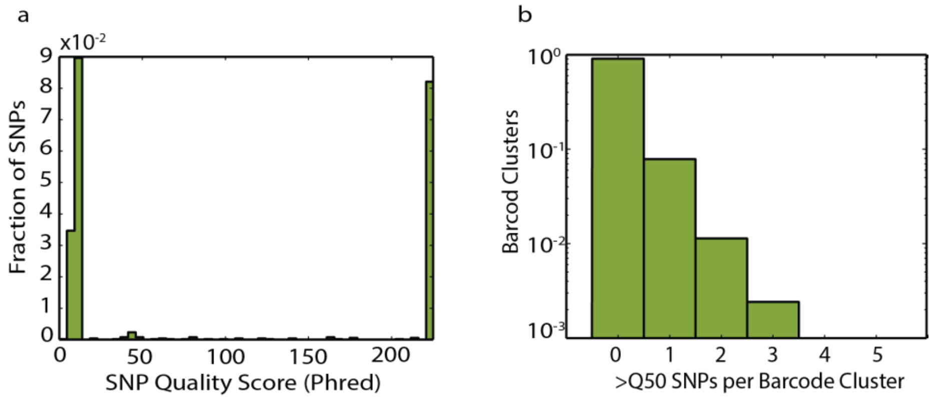

Supplementary Figure 8. Qualities and distributions of SNP calls. a) Distribution of SNP qualities scores. b) Distribution of number of high quality (>Q50) SNPs in each barcode cluster. ~90% of barcode clusters contain no SNPs while the rest are distributed into the remaining 10%.

Supplementary Table 1. Oligonucleotides used in SMDB

Oligo Name	Sequence 5'- 3	Notes	Structure
FL127	AATGATACGGCGACCACCGAGATCTACAC-TCGTCGGCAGCGTC	Primer for barcoded fragment	Illumina P5 - Nextera Const Sequence
Barcode Oligo	GCAGCTGGCGTAATAGCGAGTACAATCTGCTCTGATGCCGCATAGNNNNNN NNNNNNNNNTAAGCCAGCCCCGACACT	Barcode Oligo Sequence	ConstA-N(15)-ConstB
FL128	CTGTCTCTTATACACATCTCCGAGCCCACGAGACGTGTCGGGG CTGGCTTA	Barcode PCR Fwd	NexteraComplementary - Barcode Priming Site
FL129	CAAGCAGAAGACGGCATACGAGATCAGCTGGCGTAATAGCG	Barcode PCR Rev	Illumina P7 - Barcode Priming Site
FL166	GCCCACGAGACGTGTCGGGGCTGGCTTA	Custom Barcode Read Sequencing Primer	Contains barcode priming site of FL128
UnivAdaptorA-W	CCACTACGCCTCCGCTTTCCTCTCTATGGGCAGTCGGTGAT	Same as adaptor in NEB E6285S	Watson strand of universal adaptor A
UnivAdaptorA-C	ATCACCGACTGCCCATAGAGAGGAAAGCGGAGGCGTAGTGG*T*T	Same as adaptor in NEB E6285S	Crick strand of universal adaptor A
UnivAdaptorB-W	CCATCTCATCCCTGCGTGTCTCCGACTCAG	Same as adaptor in NEB E6285S	Watson strand of universal adaptor B
UnivAdaptorB-C	CTGAGTCGGAGACACGCAGGGATGAGATGG*T*T	Same as adaptor in NEB E6285S	Crick strand of universal adaptor B
FL178	CCACTACGCCTCCGCTTTC	Primer for Template PCR	Complementary to Adaptor A
FL179	CCATCTCATCCCTGCGTGT	Primer for Template PCR	Complementary to Adaptor B
FL143	GACCTCGCGGGTTTTCGCT	For known template library	FragC Forward Primer (5K)
FL144	CCT GAC CGC TGT ACA CTG CA	For known template library	FragC Reverse Primer (5K)
FL145	GGTGAACGATGCGTAATGTG	For known template library	FragD Forward Primer (5K)
FL146	TCAGCATCTAGCATGCAACC	For known template library	FragD Reverse Primer (5K)
FL147	TCGGATTTAGTGCGCTTTCT	For known template library	FragE Forward Primer (5K)
FL148	GCCCATGACAGGAAGTTGTT	For known template library	FragE Reverse Primer (5K)
FL170	ATTTGAATCCTCCGGCTCCG	For known template library	FragG Forward Primer (3K)
FL171	TCC CGG ACG AAC CTC TGT AA	For known template library	FragG Reverse Primer (3K)
FL172	GGC TTG GCT CTG CTA ACA CG	For known template library	FragH Forward Primer (5K)
FL173	GGA TCA GAA ATG GGA AGA AGG CG	For known template library	FragH Reverse Primer (5K)
FL174	GCC ACC TGT TAC TGG TCG AT	For known template library	FragI Forward Primer (5k)
FL175	ACC GAC TCA ATA AAC ACG GC	For known template library	FragI Reverse Primer (5k)
FL176	ACC TCT AAA TCG TGC ACA GGC	For known template library	FragJ Forward (4K)
FL177	TTC CCC GAT ACC TTG TGT GC	For known template library	FragJ Reverse (4K)

Supplementary Note 1. Droplet stability in thermal cycling

Droplet stability to thermocycling is dependent on surfactant, aqueous buffer, and droplet size. Using the EA surfactant and our PCR buffer, we found empirically that droplets are most stable to thermal cycling when they are immersed in FC-40 with 5% w/w EA surfactant and with 2% tween-20 w/v and 2% PEG-6000 w/v in the aqueous phase. Under these conditions, droplets are most stable to thermal cycling if their spherical diameter is less than 55um.

Supplementary Note 2. Algorithm to cluster error barcodes to their original sequences

The algorithm we use, called dfsCluster, available at https://github.com/AbateLab/Barcoding, operates under the expectation that each sequence in a barcode cluster is one Hamming distance away from another sequence in that cluster, and at least two Hamming distances away from any sequence not in that cluster. If this is the case, the sequences associated with unique barcode clusters form connected components in Hamming space, which can then be identified using a depth first search (dfs) in time proportional to barcode length times the number of unique sequences amongst the barcodes and their derivatives.

One scenario where this expectation doesn't hold is where sequences from one part of a barcode cluster are at least two mutations away from all sequences from another part. Computer simulation using a single length 15 template, 0.8 template replication rate, and 0.0001 single base error rate shows that although clusters do split, the splits are inconsequential. When we run dfsCluster on these simulated barcode clusters, it consistently groups 99.99% of the simulated cluster's sequences into a single cluster.

The other scenario is collisions, where multiple barcode clusters merge into a single component. Collisions are heavily dependent on the minimum Hamming distances between the original barcodes. To this end, dfsCluster does provide the option to identify and remove components suspected of being collisions from its output. This filter marks clusters where the normalized difference between the number of most, and second most populous sequences in each cluster is less than 0.7 as collisions, with a false positive rate of 0.017, and a false negative rate of 0. For more functional details, consult the Abate lab github.

Supplementary Note 3. Comparing rate of double encapsulation to theoretical Poisson distribution

If the process of template encapsulation is completely random, then the distribution of the number of templates per droplet should follow a Poisson distribution where λ is the average number of templates per droplet and k is the number of templates in a particular droplet (ie $k = 2$ represents droplets with two templates).

$$P(k, \lambda) = e^{-\lambda}\lambda^{k}/k!$$

Approximating each barcode cluster as a single droplet, the fraction of droplets that contain a single template ($k = 1$) is represented by:

$$P(1, \lambda) = e^{-\lambda}\lambda$$

The fraction of droplets containing two templates is:

$$P(2, \lambda) = e^{-\lambda}\lambda^{2}/2$$

The ratio between $P(1, \lambda)$ and $P(2, \lambda)$ is $R = 2/\lambda$. Using the number of one and two template containing barcode clusters, we estimate $\lambda = 0.1$, which matches the target encapsulation ratio and supported by

counting fluorescent vs. non fluorescent droplets in SYBR staining after initial template amplification in droplets.

Supplementary Note 4. Defining coverage entropy

In order to visualize the coverage distribution for every barcode cluster, it must be described as a numerically. We applied the informational entropy from information theory to the distribution of reads to arrive at coverage entropy *S*:

$$S = \sum_i (Pi)\left(\log\left(\frac{1}{Pi}\right)\right)$$

where *Pi* is the probability of finding a read that maps into the *i*th bin along the template in each barcode cluster. Coverage entropy is maximum when the probability for reads to fall into bin is equal.

Supplementary Methods

Gravity induced droplet size fractionation

In a tumbling emulsion, larger droplets experience higher buoyant force than smaller droplets. This phenomenon can be used in a simple method of segregating large and small droplets all in one emulsion. To use gravitational fractionation, droplets are loaded into a syringe with equal volumes of HFE7500 with 2% EA surfactant, then gently rolled along the 30° tilted long axis of the syringe at approximately 0.5Hz for one hour. The rolling fluidizes the emulsion allowing droplets to shuffle past one another based on their buoyancies. After rolling, the syringe is fully tilted to 90° facing down. The large droplets are on top of the emulsion while the small droplets are at the bottom. Half of the emulsion containing the small droplets are collected. The other half of the emulsion contains a mixture of large and small droplets which are further sorted using a pinch flow fractionation device (Fig. S3).

Simulating probability of repeating barcodes

The probability of resampling the same barcode by randomly drawing from an unlimited pool is akin to the "birthday problem" for which the analytical solution is not computationally tractable for such a large number of possible barcodes. In order to determine the probabilities, we performed *in silico* simulations. Barcodes are generated by randomly selecting one of four bases with equal probabilities until N bases are selected, resulting in a random barcode. A repeat event occurs when a newly generated barcode matches exactly with a previously generated barcode. The probability is determined by averaging the result from multiple simulations. The script used for simulation is available at the Abate Lab Github: https://github.com/AbateLab/

Calculating the limit of detection for rare variants

The probability of observing a rare mutation present at frequency *f* when sampling the population *n* times is described by a Poisson distribution:

$$P(k > 0) = 1 - e^{-(f*n)}$$

Hence, the frequency of mutants that can be detected with probability *P* is:

$$f = \frac{\ln\big(1 - P(K > 0)\big)}{-n}$$

Setting $P = 0.95$ we can calculate the minimum frequency of molecules we expect to detect 95% of the time when we sequence *n* number of molecules with SMDB.
